# Biosynthesis, Characterization, Antimicrobial and Cytotoxic Effects of Silver Nanoparticles Using* Nigella arvensis* Seed Extract

**Published:** 2017

**Authors:** Azam Chahardoli, Naser Karimi, Ali Fattahi

**Affiliations:** a *Laboratory of plant physiology, Department of Biology, Faculty of Science, Razi University, Kermanshah, Iran. *; b *Medical Biology Research Center, Kermanshah University of Medical Sciences, Kermanshah, Iran.*

**Keywords:** *Nigella arvensis*, Green synthesis, Antibacterial, Cytotoxicity, Silver nanoparticle

## Abstract

The biogenic synthesis of metal nanomaterial offers an environmentally benign alternative to the traditional chemical synthesis routes. In the present study, the green synthesis of silver nanoparticles (AgNPs) from aqueous solution of silver nitrate (AgNO_3_) by using *Nigella arvensis* L. seed powder extract (NSPE) has been reported. AgNPs were characterized by UV–vis absorption spectroscopy with an intense surface plasmon resonance band at 435 nm which reveals the formation of nanoparticles. Fourier transmission infrared spectroscopy (FTIR) showed that nanoparticles were capped with plant compounds. Transmission electron microscopy (TEM) showed silver nanoparticles, with a size of 2-15 nm, were spherical. The X-ray diffraction spectrum (XRD) pattern clearly indicates that AgNPs formed in the present synthesis were crystalline in nature. Stabilized films of exudate synthesized AgNPs were effective anti-bacterial agents. In addition, these biologically synthesized nanoparticles were also proved to exhibit excellent cytotoxic effect on a human breast cancer cell line (MCF-7) and a human colorectal adenocarcinoma cell line (HT-29). The results confirmed that the NSPE is a very good ecofriendly and nontoxic source for the synthesis of AgNPs as compared to the conventional chemical/physical methods. Therefore, *N. arvensis* seed provides future opportunities in nanomedicine by tagging nanoparticles with secondary metabolites.

## Introduction

Nanotechnology has attracted a great interest in recent years due to its expected impact on many areas such as energy, medicine, electronics, and space industries, which greatly depends on the size and shape of nanoparticles as an effect of quantum confinement of electrons. The development of new materials with nanometer size, including nanoparticles, nanotubes, nanowires, etc., is the major activity of nanotechnology (1). Nanoparticles (NPs) are being viewed as fundamental building blocks of this technology. Metal nanoparticles are extensively used in many electrochemical, electro analytical and bio-electrochemical applications owing to their extraordinary electro catalytic activity (2). Among metal nanoparticles, silver nanoparticles in particular are attracted intensive research interest of their important applications in medical industries, catalysis, and surface-enhanced scattering (3).

Recently, biological or green chemistry synthesis of NPs received enormous attention over the physical and chemical synthesis, as it is environment-friendly solvents, biocompatible and nontoxic reagents. Microorganisms, whole plants, plant tissue and fruits, plant extracts and marine algae have been used to produce nanoparticles (4-9). Plant extract mediated synthesis of metal nanoparticles has an edge over microbial mediated biosynthesis of nanoparticles because the green synthesis of nanoparticles takes place extracellular. Plant extracts may act both as reducing agents and stabilizing agents in the synthesis of nanoparticles (10). Further, this process is quick and suitable for large scale synthesis (11).

Lukman* et al*. (2011) described the AgNPs syntheses using *Medicago sativa* seed exudates, while the reduction was considered due to the phenolics present in the extract (12). Moreover, the seed extracts of various plants, including *Artocarpus heterophyllus* (13), *Jatropha curcas* (14), *Strychnos potatorum* (15),* Foeniculum vulgare* (16),* Silybum marianum* (17) and *Syzygium cumini* (18), were successfully used for AgNPs synthesis.

In this study we explore the biosynthesis of AgNPs using the grassy plant, *Nigella arvensis* belonging to the genus *Nigella* and family Ranculaceae. It is known as black seed which contain several active compounds such as different class of flavonoids, terpenoids, proteins and alkaloids (19). Black seeds have been used in public medicine as curative substances for treatment of many diseases. It has therapeutic uses like worm infestation treatment, antiallergic, antiviral & anti-inflammatory (20). Flavonoids and protein present in the plant may act as a stabilizing and reducing agent in synthesis of silver nanoparticles (21, 22). Therefore, our aim is to investigate the biosynthesis of AgNPs using aqueous extract obtained from seed of *N. arvensis*, and examine the antibacterial activity of biosynthesized AgNPs since AgNPs has been widely used as an antibacterial agent. Additionally, we have examined these biosynthesized AgNPs effects on cell viability to reflect the cytotoxicity of AgNPs against human breast cancer cell line (MCF-7) and a human colorectal adenocarcinoma (HT-29) cell line *in-vitro*.

## Experimental

*Materials*

AgNO_3_ (99.98%) and nutrient agar (Merk) was purchased from Shimigostar Company (Kermanshah, Iran). 3-(4, 5-di-methyl-2-thiazolyl)-2,5-diphenyl-2H-tetrazolium bromide (MTT) 2-7-diacetyl dichlorofluorescein (DCFH-DH), trypsin and DMSO were purchased from Sigma–Aldrich, USA.

*Collection of plant materials*

The seeds of *N. arvensis* were purchased from Pakan Bazr Company in Isfahan, Iran. The seeds were rinsed with water thrice followed by Milli-Q water to remove the fine dust materials and then, the seeds were air dried for 1 week to remove the moisture completely.

*Seed exudate preparation*

20 g of dried *N. arvensis* seeds powder was added to 100 mL ultrapure water in 250 mL Erlenmeyer flask. The mixer was boiled (15 min), cooled, filtered, and the filtrate was stored at 4 °C as the stock solution and was used within 3 days. 

*Synthesis of AgNPs*

For synthesis of AgNPs, the 1 mL of aqueous filtrate extract of *N. arvensis* was taken into 100 mL of Erlenmeyer flask. Then the extract was mixed into silver nitrate (AgNO_3_) to make the final volume concentration of 1 mm solution. The reaction mixture was exposed to sunlight irradiation condition until the color change was arisen. Then, the reaction mixture of seed extracts and AgNPs was subjected to centrifugation at 8,000×g for 15 min; resulting pellet was washed three times with deionized water and filtered. An aliquot of this filtrate containing AgNPs was used for FTIR, XRD and Transmission Electron Microscope (TEM) analysis.

*UV–Visible Spectrometry*

The end point of reaction was evaluated by UV–vis spectroscopy. The biologically reduced brown color solution mixture was scanned by Shimazdu, Lambda UV mini-1240 instrument operated at a resolution of 1 nm. The UV–visible analysis was performed in the absorption wavelength of 200–700 nm periodically for 1 h. order to observe rapid reduction AgNPs by the action of plant extracts. The distilled water was used as a blank. 

**Figure 1:**
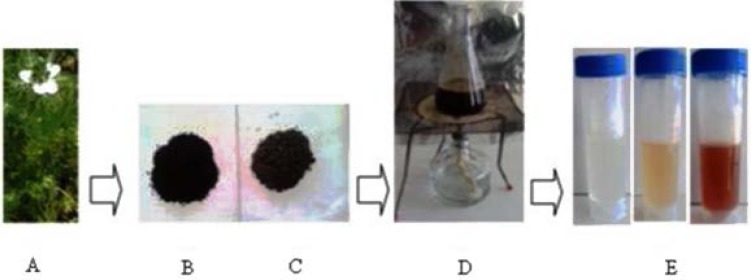
Biosynthesis of silver nanoparticles from seed aqueous extract of *N. arvensis*

A) Plant B) Seed C) Powder D) Extraction E) Biosynthesized nanoparticle.

**Figure 2:**
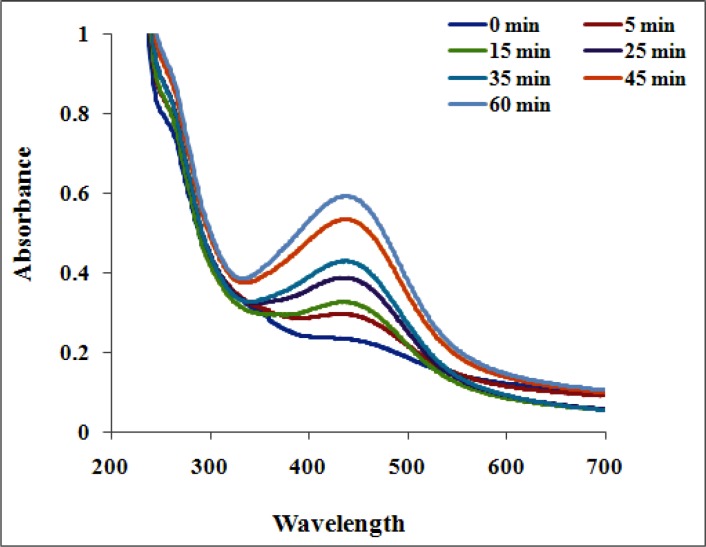
UV–visible spectrum of biosynthesized AgNPs showed peak at 435 nm

**Figure 3:**
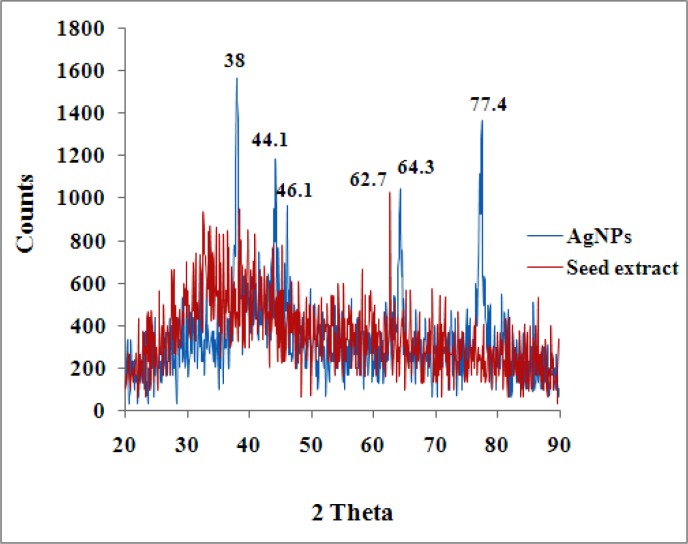
XRD spectrum of silver nanoparticles and seed aqueous extract of *N. arvensis.*

**Figure 4:**
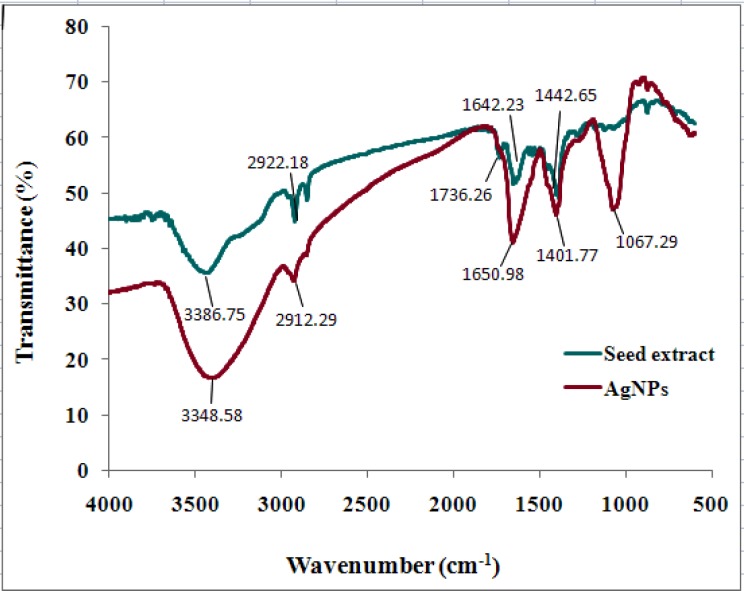
FTIR spectrum of seed extract of *N. arvensis* and synthesized AgNPs.

**Figure 5.**
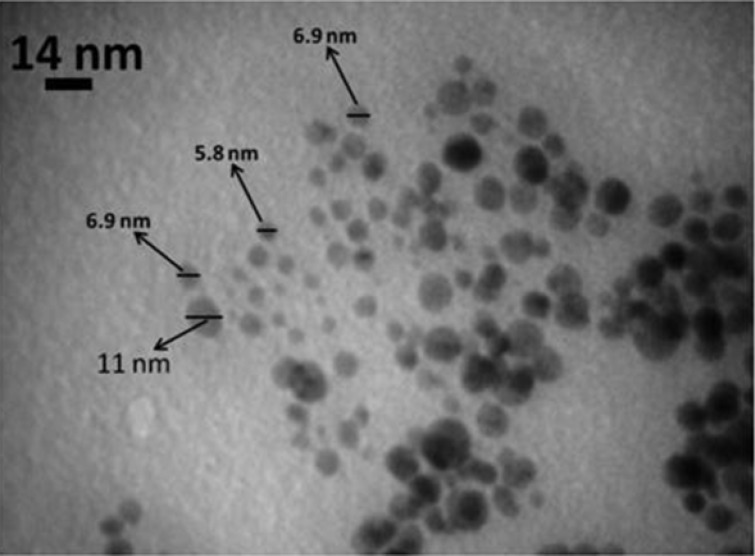
TEM micrograph of AgNPs synthesized from seed aqueous extract of *N. arvensis*

**Figure 6:**
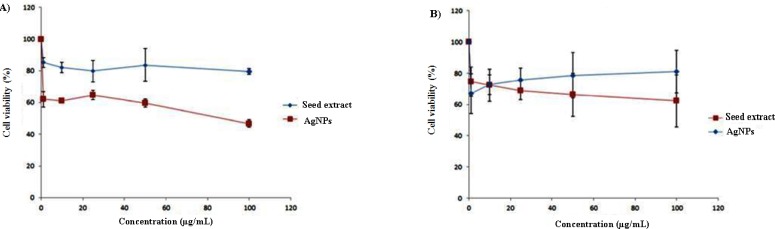
Cytotoxicity assay – cell viability of A) HT-29 and B) MCF-7 cells exposed to different concentrations of biosynthesized AgNPs

**Table 1.** Inhibition zone (mm) of biosynthesized AgNPs using* Nigella arvensis* against various microorganisms

**Microorganism**	**Gram nature**	**Inhibition zone diameter (in mm)**		
		**Extract** ** (50 µL)**	**AgNP** ** (** **20 µL)**	**AgNP ** **(50 µL** **)**
***Streptococcus pyogenes***	Positive	10	11	17
***Bacillus subtilis***	Positive	14	13	14
***Staphylococcus aureus***	Positive	8	12	15
***Escherichia coli***	Negative	10	10	11
***Proteus mirabilis***	Negative	11	11	21
***Salmonella typhimurium***	Negative	10	12	15

*Characterization of silver nanoparticles*

The synthesized AgNPs were characterized using FTIR, XRD and TEM. The FTIR is performed to the extract which was exposed before and after addition to the AgNO_3_ solution. The samples were mixed with KBr to make a pellet and it was placed into the sample holder. The spectrum was recorded at a resolution of 4 cm^−1^. 

For XRD studies, dried nanoparticles were coated on XRD grid and the spectra was recorded by using APD 2000- Italian structures X-ray generator operated at a voltage of 40 kV and a current of 30 mA with Cu K^-1 ^radiation.

The morphological analysis of the particle was done with TEM. The drop of aqueous AgNO_3_ sample was loaded on carbon-coated copper grid, followed by solvent evaporation at room temperature for an hour. The TEM micrograph images were recorded on Zeiss - EM10C instrument on carbon coated copper grids with an accelerating voltage of 80 kV. The clear microscopic views were observed and documented in different range of magnifications.

*Antimicrobial activity*

The antibacterial activity was assayed by well diffusion method. The synthesized AgNPs antibacterial activity was observed against pathogenic strains of bacteria such as *Staphylococcus aureus*, *Streptococcus pyogens*,* Bacillus subtilis*, *Escherichia coli*, *Proteus mirabilis* and* Salmonella typhimurium*. The bacterial suspension was swabbed on the nutrient Agar (NA) plates using sterile cotton swab. 20 µL and 50 µL of AgNPs prepared from seed extract were added into the wells with 6 mm diameter. The seed extract of *N. arvensis* (50 µL) was used as a control. The plates were incubated at 37 °C for 24 h. Positive test results were scored when a zone of inhibition was observed around the well after the incubation period.

*Cytotoxicity studies*

The cytotoxicity studies of biosynthesis AgNPs were performed on a human breast cancer cell line (MCF-7) and a human colorectal adenocarcinoma (HT-29) cell line using 3-(4,5-dimethylthiazol-2-yl-)-2,5-diphenyltetrazolium bromide (MTT) assay. Briefly, 180 µL of cell suspension at a concentration of 5×10^4^ cells/mL was seeded on 96-well plates for 24 h. before the assay. Following 24 h. growth, 20 µL of NP suspension, which was freshly prepared at varying concentrations in distilled water was added into each wells. Distilled water was used as control. After 48 h. cells were rinsed, and 100 µL of fresh medium containing 0.5 mg/mL of MTT was added to each well. Plate was incubated for an additional 3 h. Each well was then washed with 50 mL of PBS after the medium containing the unreacted MTT was removed. Then, 150 mL of DMSO was added to each well to dissolve the formazan crystals. Finally, the absorbance was determined on an ELISA plate reader (Bio-Rad, Model 680, USA) with a test wavelength of 570 nm and a reference wavelength of 630 nm to obtain sample signal (OD570–OD630). All conditions were done in six triplicate in two independent experiments for each cell line. 

The cell viability was expressed as follows;

Viability (%) = A_s_/A_c_×100

Where A_s_ and A_c_ are the mean absorbance of AgNPs treated and control cells respectively.

## Results and Discussion

Formation of AgNPs by the reduction of AgNO_3_ during treatment with the seed extracts of *N. arvensis* is evident from the change in color of the reaction mixture which turned yellow to brown by exposing to sunlight (Figure 1). The appearance of the brown color was due to the excitation of the surface plasmon vibrations absorption spectrum of seed extracts at different wavelengths ranging from 300 to 700 nm revealed a peak at 435 nm (23). The surface plasmon resonance (SPR) band is influenced by size, shape, morphology, composition and dielectric environment of the prepared nanoparticles (24). The UV–Vis spectra recorded from the *N. arvensis* reaction vessel at different times and OD value are plotted in Figure 2. Silver nanoparticles exhibit unique and tunable optical properties on account of their surface plasmon resonance, dependent on shape and size distribution of the nanoparticles stabilizing molecules or the surface adsorbed particles and the dielectric constant of the medium (24, 25). Previous studies have shown that the spherical AgNPs contribute to the absorption bands at around 400 nm in the UV–vis spectra (26, 27).The SPR band characteristics of AgNPs were detected around 435 nm (Figure 2). This is strongly suggests that the AgNPs were spherical which have been confirmed by the TEM results of this study.

The characteristics peak observed in X-ray diffraction pattern of the biosynthesized AgNPs produced by the seed of *N. arvensis* extract further demonstrated and confirmed the presence of AgNPs (Figure 3). The XRD peaks at 38°, 44.1°, 46.1, 64.3 and 77.4° can be indexed to the (111), (200), (220) and (311) planes for silver respectively. It suggests that the prepared AgNPs biphasic in nature. Similar report for XRD has shown in *Eclipta prostrate, Tribulus terrestris* and *Prosopis juliﬂora* extract for synthesized AgNPs (24, 28, 29). The resultant XRD spectrum clearly suggests that the AgNPs synthesized from the seed extract of* N. arvensis* was crystalline which strongly proves in TEM micrograph images (Figure 3).

FTIR analysis was used to characterize and identify biomolecules that were bound specifically on the synthesized AgNPs. The biologically synthesized AgNPs and the powdered seeds were mixed with the Potassium bromide to make a pellet. The FTIR spectra of control dried *N. arvensis* seed extract (before reaction without AgNO_3_) and synthesized AgNPs (after reaction with AgNO_3_) are shown in Figure 4. 

FTIR results indicate that absorption bands at 3348 (O–H stretching, H–bonded of alcohols, phenols and N–H stretching of primary, secondary amines, amides of protein), 2912 and 2846 (C-H stretching of alkanes), 1650 ( -C=C- stretching and N–H bend of alkenes and primary amines), 1401 (C-C stretching (in- ring) of aromatics and 1067 (C-O stretching of alcohols, carboxylic acids, esters and ethers and C–N stretching of aliphatic amines). Both of them showed a shift of the absorption bands of 3386 to 3348, 1736 to 1650, 1643 to 1401 and 1442 to 1067 cm^-1^ after bioreduction that vibrational bands corresponding to bonds such as –C= C- and –C= O are derived from the compounds such as flavonoids and alkaloids in *N. arvensis* seeds. So it is assumed that these biomolecules and some proteins are responsible for capping, stabilization and reduction of Ag^+^ to AgNPs.

The FTIR analysis indicated the involvement of amides, alkanes, carboxyl, alcohols and phenolsgroup presented in the synthesized AgNPs. A similar observation is noticed in biological synthesis of AgNPs using *Artocarpus heterophyllus* Lam. (13) and *Abelmoschus esculentus* (28) seed extract.

The biologically synthesized AgNPs using the seed extract of *N. arvensis* structural morphology and crystallinity were further confirmed by TEM micrograph images (Figure 5). The TEM micrograph image of synthesized AgNPs was observed in spherical shape and the average size of 8.5 nm. According to the results showed in Figure 5, control of the size and morphology of AgNPs can be related to the interactions between reducing biomolecules like terpenoids, alkaloids and flavonoids and metal atoms (28, 29). It was noticeable that the edges of the particles were lighter than the centers, suggesting that biomolecules, such as proteins in plant capped the AgNPs. It is seen that proteins are present among the particles and are adhered to their surfaces (13, 30).

Previous studies have shown that small spherical Ag nanocrystals will exhibit a single SPR band, while anisotropic particles will exhibit two or three bands depending upon their shape. Larger particles will in turn exhibit other bands related to quadrupole and higher multi-pole plasmon excitation (31). The formation, shape, size, and distribution of nanoparticles is depending on physiochemical properties such as temperature, time, pH, optical and concentration of the substrate (32).

Plant extracts are contain biomolecules (such as phenolics, terpenoids, polysaccharides, flavones, alkaloids, proteins, enzymes, amino acids, and alcoholic compounds), which act as both reducing and capping agents that form stable and shape-controlled nanoparticles (33). The concentrations of plant extracts (aqueous or alcoholic extracts) play an important role in maintaining the shape and size of the nanoparticles (34). The increase in the concentration of the plant extract will also increase the absorbance intensity and the surface Plasmon peak is slowly shifted toward lower wavelength at high concentrations that may be due to blue shift and depends on the particle size and shape (35). These studies in TEM have shown that the presence of a capping layer in plant mediated synthesis of AgNPs, where the plant extract acts as capping layers, shapes the nanoparticle during its growth (33).

The antibacterial activity of the synthesized NPs was evaluated against gram positive (*Streptococcus pyogenes*, *Bacillus subtilis*, and *Staphylococcus aureus*) and gram negative (*Escherichia coli*, *Proteus mirabilis*, *Salmonella typhimurium*) bacteria at different concentrations (Table 1) and it was observed that increase in concentration of NPs progressively inhibit the growth. The gram negative bacterium *P. mirabilis* showed maximum zone of inhibition at 21 mm which may due to the presence of peptidoglycan layer in gram positive bacteria thus forming more rigid structure leading to difficult penetration of the AgNPs compared to the gram negative bacteria where the cell wall possesses thinner peptidoglycan layer (34). 

The antibacterial activity of AgNPs synthesized using *Artocarpus heterophyllus* seed extract (13)*, Tribulus terrestris* (24).* Boswellia ovalifoliolata, Svensonia hyderobednesis* and* Shoreatum buggaia *leaves extract (36), also showed a similar result. The higher bacterial activity was certainly due to silver cations released from AgNPs as negatively charged bacterial cell wall is more attracted to silver ions causing bacterial cell wall rupture and finally cell death (25, 37, 38). 

Previous studies indicated the antibacterial activity of AgNPs by attachment to the bacterial cell wall, or the formation of free radicals (39, 40). In addition, the silver ions released from AgNPs may play a vital role of the antibacterial activity due to the interaction of silver ion with the thiol groups of enzymes (41). Furthermore, it was shown that the antibacterial activity of AgNPs was size and shape dependent. AgNPs (1–10 nm) attach to the surface of cell membrane and drastically disturb its proper function like respiration and permeability (42).

There are several studies indicated that AgNPs are cytotoxic, and their cytotoxicity is size and dose dependent. It has also been shown that surface modifications of AgNPs can dramatically alter the toxicity (43-46). On the other hand, there has been a great interest in the use of natural compounds for the treatment of cancers. A multitude of medicinal herbs have the anticancer properties (47). A study at 2007 has indicated that among 155 FDA-approved small molecule anticancer drugs, 47% were either natural products or analogues inspired by them (48). Therefore, the synergistic effect of herbal extract and AgNPs can be a promising strategy for cancer therapy. Green synthesized AgNPs were evaluated for potential antitumoral cytotoxicity against human MCF-7 and HT-29 cell lines under MTT method. In both of cell lines, seed extract was not toxic at concentration used in this study, and only 20-30% proliferation inhibition was achieved. MTT reduction by HT-29 cells significantly decreased after a 48 h. treatment with 1 μg/mL or higher doses of AgNPs in a dose-dependent manner (Figure 6, *P* < 0.05) and the IC_50_ of AgNPs was calculated to be around 100 μg/mL. In the recent study, chitosan coated AgNPs had effective cytotoxicity on HT-29 while bare NPs cytotoxicity was significantly lower (49) which indicates cytotoxicity of AgNPs is significantly affected by coating material and environment of NPs. AgNPs treatment also inhibited MTT reduction by MCF-7 cells but its cytotoxicity effect was lower than cytotoxicity observed in HT-29 and no significant difference was observed between extract and AgNPs. Therefore, cytotoxicity of green synthesized AgNPs is dependent on cell type that indicates a specific intra cellular mechanism for proliferation inhibition rather than unspecific disruption of cell membrane functionality. 

## Conclusion

The present study described a green, eco-friendly and simple way to synthesize AgNPs by* N. arvensis* seed extract. This is a simple, green and efficient method to synthesize AgNPs at room temperature without using any harmful reducing agents.The green synthesized AgNPs were characterized using UV–visible Spectroscopy, XRD Spectrum, FT-IR Spectroscopy and TEM analysis. The synthesized AgNPs was spherical crystalline structure, 2–15 nm in size, with functional groups from the seed extract capped on the surface. The synthesized AgNPs using seed extract of *N. arvensis* proved antimicrobial activity against clinically isolated gram positive and gram negative resistant human pathogens. Furthermore, in the present study AgNPs exhibited a significant cytotoxic effect on MCF-7 and HT-29 cell lines.
